# Shifting Skills in Robot-Assisted Surgery: Case Study With Interdisciplinary Perspectives for Human Factors and Career Research

**DOI:** 10.2196/87735

**Published:** 2026-09-22

**Authors:** Thomas Ellwart, Julia Birke, Johanna T Paul

**Affiliations:** 1 Department of Business Psychology, Trier University, Universitätsring 15, Trier, Rheinland-Pfalz, 54296, Germany, 49 6512013051

**Keywords:** delivery of health care, nontechnical skills, technical skills, robotic surgical procedures, sustainable career, growth needs

## Abstract

**Background:**

The introduction of STARA (smart technology, AI, robotics, and algorithms) changes work processes and the application of skills, depending on specific task design within a given context.

**Objective:**

From a Human Factors perspective, this study uses empirical data from a case study to demonstrate how different task designs influence the application of theater nurses’ skills during robot-assisted surgeries. Moreover, from a transdisciplinary perspective, the data elucidate that STARA-related task design extends beyond Human Factors’ perspectives, encompassing cross-level effects between task configurations of different surgical units and individual evaluations regarding career motives. Finally, the study aims to integrate interdisciplinary perspectives by outlining theoretical, methodological, and practical implications for both STARA-related Human Factors task design in health care and Sustainable Career Research.

**Methods:**

Empirical data derive from an embedded single-case study design within a hospital. The case study illustrates field experiences from a focus group interview (n=5) and postsurgery surveys (n=36) on Human Factors–related variables (objective task variety, perceived workload, perceived application of professional skills), and variables from Career Research (perceived career sustainability, growth needs).

**Results:**

Higher task variety and skill application during surgeries correlate with long-term career sustainability evaluations. Moreover, career-related growth needs relate to interindividual differences in STARA task evaluations. Interdisciplinary perspectives with respect to theoretical models, practical implications, and methodological challenges are discussed.

**Conclusions:**

The introduction of STARA (smart technology, AI, robotics, and algorithms) changes work processes and the application of skills, depending on specific task design within a given context.

## Introduction

### Background

**Figure 1.**
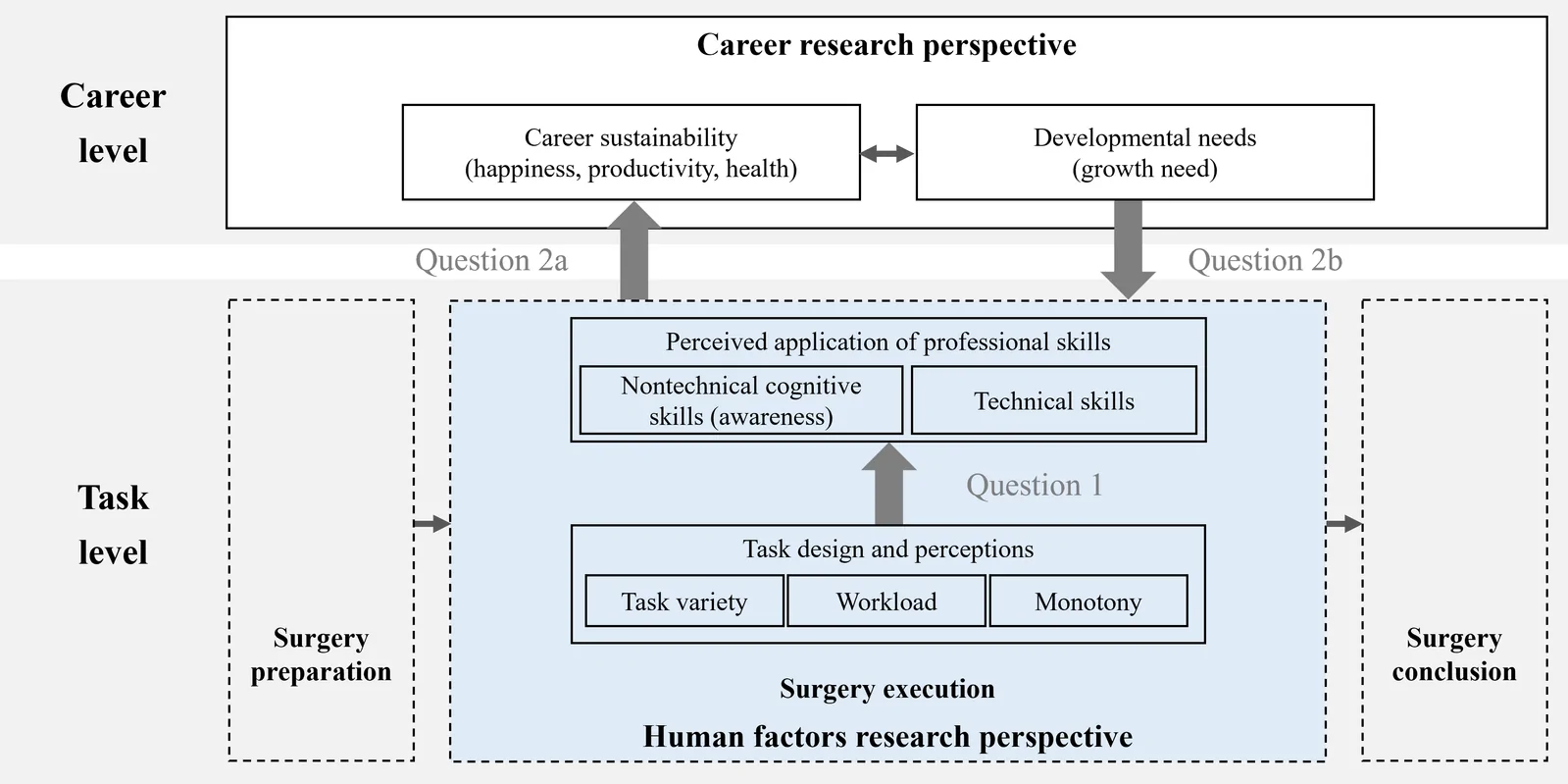
Task and career level perspectives of theatre nurses in RAS.

The implementation of smart technologies, AI, robotics, and algorithms (smart technology, AI, robotics, and algorithms; STARA) has substantial interdisciplinary implications in the health care sector. In Human Factors, the focus lies on designing work systems that are human-centered, efficient, effective, and resilient. In the field of human resource management, the emphasis is, for example, on questions of sustainable career development. One prominent example, among many, is the da Vinci Surgical System (Intuitive Surgical Inc), which has significantly transformed the landscape of surgery and, in particular, the role of operating room nurses who assist surgeons during procedures [1]. During surgical execution, the surgeon operates the robotic system from a console, using hand controls to manipulate instruments while viewing the surgical field in detail. However, the surgeon no longer stands face-to-face at the table with the theater nurse and does not interact directly. As a result, nurses’ tasks are modified, and task designs can differ across units within a hospital. This results in a varying number of tasks that nurses perform during robot-assisted surgery (RAS), thereby impacting workload, monotony, and the application of professional skills during these tasks. In that respect, the project underpinning the case study focused on a Human Factors perspective to elaborate on the following research question: “How are different task-specific demands in surgical workflows related to perceived skill application?” (see Question 1 in Figure 1). Specifically, nontechnical cognitive skills were investigated to predict safety and resilience of the work-system [2,3]. However, expanding the extensively studied Human Factors perspective, technological work design goes beyond the task-specific focus. Human resource-oriented career research, for instance, examines the effects of technological advancements on career intentions, yet typically does not incorporate the task-specific theoretical and methodological approaches established in Human Factors Research. This gap reveals considerable interdisciplinary potential between Human Factors and Career Research and may be illustrated by quotes from preparatory interviews with nurses in this study. These interviews indicated that changes in skill application during surgery influence individual career evaluations and, consequently, the sustainability of career projections. As one participant noted, “I did not choose a career as a theater nurse to be bored by watching robotic arms during surgery,” summarizing this perception. Against this background, the present study goes beyond the Human Factors perspective and questions “How do task-specific demands and perceived skill application align with reflection of career sustainability?” and vice versa “How do career-related needs relate to situational evaluations of task-specific demands and perceived skill application?” in order to illustrate the relationship between task-level design of robot-assisted surgeries and nurses’ career evaluation (see Questions 2a and 2b in Figure 1).

Overall, this study seeks to empirically capture and conceptually discuss the interdisciplinary potential between Human Factors and Career Research, using the example of RAS. Career Research investigates the changing work tasks and skill requirements within the STARA environment [4-6]. Reflecting the career-related experiences of theater nurses in the case study, we focus on the concept of career sustainability [7]. Thus, we aim to integrate task-level concepts from Human Factors into models of Career Sustainability, emphasizing the potential for an interdisciplinary examination.

### Study Aims

#### Empirical Human Factors Perspective: Task Design and Skill Application in RAS

Using exploratory data from a case study, we aim to examine, from a Human Factors perspective, how different task-specific demands in surgical workflows of RAS are related to perceived skill application. We explore task-specific concepts from Human Factors Research within a socio-technical environment, highlighting functional task design as essential for ensuring human-centered work systems [8,9]. This focus includes task-related variables such as task variety, perceived workload, and monotony, alongside the perceived application of professional skills during surgical situations (Question 1).

#### Empirical Transdisciplinary Perspective: Human Factors and Career Evaluation in RAS

Through a case study, we aim to illustrate that STARA-related changes in tasks of theater nurses (eg, surgery execution) go beyond Human Factors’ questions and address cross-level effects between task-level related changes and individual perceptions at career level (Question 2). In a field sample, we explore nurses’ reflections on career sustainability after RAS with different degrees of task variety and skill application (Question 2a). Vice versa, the influence of individual career-related needs on the Human Factors’ variables is explored with respect to interindividual differences in task evaluation (Question 2b).

#### Interdisciplinary Perspectives: Theoretical, Methodological, and Practical Implications

Following the exploratory case study, theoretical, methodological, and practical implications for Career Research and Human Factors are presented. The data collected contributes to a better understanding of the impact and potential effectiveness of career development and workplace interventions. In 5 implications, we elaborate on the addition of variables at the task-level in existing frameworks for sustainable careers, reflect approaches to human-centered work design, and discuss methodological issues.

The subsequent section briefly introduces the theoretical key concepts from Human Factors and Career Research to elaborate the empirical research questions. It then outlines the study’s contribution, followed by a presentation of the empirical study in the method and results sections and, in the discussion, a more in-depth reflection on the interdisciplinary perspectives.

### Theoretical Context

#### STARA and Human Factors Research

##### Overview

Since the 1980s, the “ironies of automation” have been a central topic in the multidisciplinary field of Human Factors [10]. Instead of simplifying tasks, automation can critically change the demands placed on human operators by reducing active application of skills, leading to more passive tasks such as monitoring [8]. Higher automation levels can diminish valuable hands-on expertise due to limited engagement with manual tasks, which can in turn lead to deterioration of skills required for problem-solving and quick decision-making in high-stakes situations [2,11]. This issue becomes more pronounced with the rise of STARA, where technology interacts interdependently with individual workers or teams–in industry as well as in health care [12]. Subsequently, the need for designing automated work environments which balance technological support with opportunities for skill application and development becomes especially important, as system resilience relies heavily on the operator’s situational awareness (SA) adaptability and skill application [13]. In this study, RAS serves as an empirical example for the shift in professional skills due to the introduction of a STARA-technology. Its introduction into surgical practice redefines work and task processes, disrupts established workflows, and necessitates the redistribution of primary and secondary tasks between technology and professional staff [11,14]. But does technology such as RAS adequately represent the broader concept of STARA? In this study, we use the term STARA to refer to the broader category of smart technologies, AI, robotics, and algorithms that are not just automations of manual work but are characterized by different Levels of Autonomy (LoA) [11].

##### Level of Autonomy in STARA Environments

RAS represents a specific type of STARA, as it mediates human work through a semiautonomous technological system embedded in a complex, multiteam environment [15]. Current generations of da Vinci systems are positioned at lower LoA (eg, level 1) [16,17], functioning in a master-slave configuration and therefore unable to operate autonomously. Nevertheless, underlying AI and algorithms have supportive functions in enabling the system to execute essential secondary tasks, such as image stabilization, motion scaling [15], and augmented reality overlays integrating preoperative imaging data into the live surgical view [18-20]. Following Endsley [11], current RAS systems would be positioned at a relatively low level of executive task support. Determining the level of autonomy at the task level is crucial for comparing the effects of different STARA technologies in health care. Existing frameworks provide useful distinctions between different task types (eg, monitoring, decision-making, and execution) and their respective levels of autonomy, thereby enabling a more nuanced assessment of technological capabilities and human-autonomy interaction [15,21].

The introduction of RAS technology in particular, and STARA technologies more generally, alters the primary tasks of nurses; however, changes in subsequent task characteristics such as variety are driven not only by the LoA of the technology itself but also by the technology-independent design and allocation of secondary tasks. In the present study, surgical teams applied the same RAS system differently, which led to varying task demands for the nurses. The following sections examine how technology-induced changes in task-specific demands affect skill application and broader career-related outcomes in health care settings.

##### Task-Specific Demands

A central focus of Human-Factor-oriented work design is the work-task itself, with one of its core constructs being task variety. Task variety is defined as the number of distinct tasks an operator must perform within a given job [22]. Research has shown that higher task variety is associated with subjective performance, job satisfaction due to more stimulating and interesting tasks, reduced monotony, increased mental alertness, and greater engagement [8,23]. In STARA environments, technology can take on tasks which were previously performed by humans and thereby reduce task variety [1]. These STARA-related task changes will influence another core construct, namely perceived workload. More specifically, during a RAS with reduced task variety, theater nurses perceive less workload than during non-RAS procedures [24,25]. In practice, however, whether a decrease, invariance, or even increase of task variety and workload will occur after the introduction of RAS depends on unit-specific work design and (secondary and primary) task allocations to the theater nurses [25]. If tasks are delegated to technology without compensatory task design, this subsequently results in decreased involvement of nurses and relates to higher monotony or lower application of professional skills. The latter phenomenon represents the key difference between surgical teams in this study, which was investigated with respect to the effects on professional skill application.

##### Technical and Nontechnical Professional Skills and System Resilience

In this paper, we use the umbrella term of professional skills and differentiate technical and nontechnical skills [3]. Technical skills often refer to trained knowledge and behaviors required to perform specific tasks. They are present across situations, acquired through training, and form an essential element of an individual’s professional competencies [26]. Additionally, Human Factors also considers nontechnical skills, which refer to more generic behavioral, emotional, or cognitive activities shown in a specific situation [27]. Nontechnical skills can further be divided into two subgroups: cognitive (eg, SA) and social (eg, teamwork) nontechnical skills [27].

Nontechnical cognitive skills are significant predictors of safety and resilience in socio-technical work systems [2,3]. In particular, the nontechnical skill of SA is defined as a 3-level hierarchical construct. It consists of the perception of information and stimuli from the environment (I Perceiving), the understanding of the meaning of this information and stimuli (II Understanding), and the anticipation of their state in the future (III Projecting) [28,29]. With a high level of SA, any changes in a situation can be noticed and, for example, communicated to the team [30], which highlights its importance for team resilience. Regarding the health care sector, maintaining SA or its levels can prevent errors in the work process, for example, by recognizing changes in vital signs, thus ensuring patient safety [29,31]. SA is, therefore, primarily intended to prevent critical situations from arising or escalating [32] and is of utmost importance in maintaining resilient systems [2].

From a Human Factors perspective, the effects of unit-specific task design and its effects during surgeries were of interest. We evaluated how task demands (ie, objective task variety, subjective workload, and perceived monotony) during RAS surgeries influence nurses’ application of professional skills (Question 1).

### Sustainable Careers: Task Demands, Professional Skills, and Needs

The case of RAS underpins the need for a trans- and interdisciplinary approach: at the task-level, it focuses on Human-Factors questions about optimal and resilient work design. At the career level, it raises pivotal issues of how technologically driven change shapes long-term professional identities and career evaluations. Whilst the former sections examine these interdependencies by analyzing task-specific demands and skill application, the following section focuses on the broader consequences for career sustainability in STARA-mediated health care settings.

#### Concepts and Models of Sustainable Careers

Most scholars define sustainable careers as “the sequence of an individual’s different career experiences, reflected through a variety of patterns of continuity over time, crossing several social spaces, and characterized by individual agency, herewith providing meaning to the individual” [33]. More specifically, the theoretical model of De Vos et al [7] provides three key indicators that distinguish sustainable from unsustainable careers, namely health, happiness, and productivity. Crucial for perceived sustainable careers is a dynamic person-career fit over time, described as the ability to adapt and evolve with changing needs and environmental demands [34]. Chin et al [5] differentiate four dimensions (resourceful, flexible, renewable, and integrative) to capture the dynamic nature of career sustainability in fast-paced environments. The dimension of “renewability” highlights task redesign due to STARA, requiring employees to reorient their role perceptions, knowledge, and skills which also applies to the example of RAS as explained below.

#### Professional Skills and Task-Specific Design

On a task-level, “renewability” implies that workers evaluate the opportunities to apply, update, or learn role-related professional skills in the light of STARA task-change. In our case study, da Vinci robots fundamentally change the way theater nurses can participate in the workflow. A perceived loss of role-identity creating activities on task-level should therefore influence their evaluations on the career level. If the nurses are hindered in applying their professional skills during the surgery and anticipate this particular task design as a future scenario, they might experience a sense of role incongruence and frustration, leading to the anticipation of a less sustainable career.

However, Chin et al [5] do not elaborate or define specific task-level variables in their model on sustainable careers. Other literature on Career Research has differentiated between tasks within a job [6] or followed a task-based approach to investigate the effects of automation on skill requirements [35]. However, despite the detailed listing of tasks there is a lack of linkage, for example through integration into subsequent processes and analysis of preliminary and secondary tasks. Furthermore, the measurement of skills or competencies tends to be on a much more generic level in studies of Career Research [36,37]. Human Factors, on the other hand, investigates perceived application of professional skills on a “fine-grained” task-level of analysis in specific contexts, which could add to the understanding of career sustainability evaluation and model development.

Complementing the antecedents of sustainable career development in the model by Chin et al [5], a task-level perspective (task variety, workload, or complexity) may contribute to the individual and organizational level of the present theory. To our knowledge, no studies have been conducted on this cross-level mechanism between variations in the task-specific design of a use case and long-term evaluations of career sustainability. Thus, we explore in the case study how task-specific demands and perceived skill application align with reflections on career sustainability (Question 2a).

#### Career-Related Needs and Situational Perception of Workload and Skill Application

Several researchers elaborate on individual needs or motivation with respect to career evaluation. For example, Chin et al [5] point out that the needs of workers keep changing over their life courses, leading to interindividual differences in desires for sustainable careers. De Vos et al [7] indicate that the fulfillment of personal needs contributes to perceived career satisfaction or success. Especially, the sustainability indicator of happiness relates to how well the career aligns with one’s values and needs, for example, the need for personal growth [7,38]. The opportunity to develop and apply new skills contributes to perceived growth and is also considered a facet of subjective career success [39]. In contrast, frustration through a mismatch between personal career needs and developmental opportunities has been linked to higher turnover intentions and job dissatisfaction [40]. However, career-related needs have not yet been connected to evaluations of a specific STARA-related task change. For example, workers with a high need for personal growth would evaluate tasks more critically if STARA hinders the application of skills rather than fostering development. On the other hand, workers with low growth needs would evaluate the objectively similar task less critically and therefore differ from their colleagues within the same unit. Thus, we explore in the case study how career-related needs correspond to situational evaluations of workload and skill application (Question 2b).

### Study Contribution

The contribution of this paper is threefold. Firstly, the transdisciplinary multilevel perspective (person, task, and organization) enriches conceptual frameworks for both STARA-related Career Research and Human Factors. Career Research may benefit, for example, if taxonomies or task-level concepts of STARA design from Human Factors are integrated into career-related models to allow differentiated predictions on job threats and career decisions. Conversely, Human Factors will benefit from Career Research if task-designers recognize effects of interindividual differences in career-related variables on the evaluation of STARA design.

Secondly, the paper elaborates on theoretical and methodological perspectives for interdisciplinary STARA research. For example, the variance of task variety, workload, or skill-application between two embedded units raises questions of the appropriate levels of analysis for research models predicting career evaluations.

Thirdly, the paper contributes to applied questions of career development and Human Factors design on multiple levels. Organizations in health care and other STARA-affected fields (macro-level) are urgently looking for highly committed professionals (microlevel) for jobs in a drastically changed task-environment (meso-level). Thus, workplace interventions addressing task design and the consideration of interindividual differences (eg, growth needs) will contribute to retaining specialists in key professions with STARA work tasks that promote both the desired efficiency gains in productivity and personal development goals.

## Methods

### Background and Setting of the Case Study

In the project underpinning the case study, da Vinci assisted surgery was applied in two units (urological and visceral surgeries) of a hospital. Despite the similar surgical context, the number of tasks (task variety) for theater nurses differed between operating units due to unit-specific task design. Thus, this setting can be classified as embedded (multiple units) single-case design [41] and is illustrated through qualitative and quantitative data.

The underlying project had an application-oriented Human Factors character and aimed to explore qualitatively and quantitatively the effects of different task-specific designs in surgical workflows. A qualitative semistructured focus group interview (n=5) was conducted prior to the quantitative data collection (n=36). The aim of the interviews was to gain a qualitative understanding of the tasks and challenges during RAS and to explain the planned quantitative data collection in the units. Extending beyond a Human Factors focus, career-related experiences were also reported in these interviews. Both perspectives, Human Factors and Career, were subsequently examined empirically and exploratorily in the quantitative phase. This quantitative part of the case study was not designed as a hypothesis-testing study but provides insightful transdisciplinary perspectives.

### Methods

#### Part One: Semistructured Focus Group Interview

The qualitative interview data of the case study derived from a semistructured focus group [42] involving five nurses experienced in the da Vinci X Surgery System (Intuitive Surgical Inc). All had a background in urology, with four also having experience in visceral surgery. The group included two operating department practitioners and three theater nurses. The focus group took place in a room adjacent to the operating theater of the hospital featured in this case study. The Human Factors question (Question 1) prompted was “What changes does the da Vinci robot cause in the different phases of surgery compared to laparoscopic surgery?” During the focus group interview, various aspects were discussed, such as changes in team collaboration (processes), changes in one’s own work and role (tasks, requirements, and skills), occurrence and management of problems, disruptions and critical situations, as well as general concerns, wishes, or ideas for the future design of RAS. Statements from the in-depth focus group interview were documented through field notes and assessed using thematic analysis [43] but without an elaborate coding strategy. Results are reported in a thematic structure later in this paper to illustrate experiences of nurses in terms of Human Factors and career-related perspectives in line with the research questions formulated above. Career-related aspects were not directly elicited but were reported by participants as outcomes of task-related work design and were further explored in the quantitative part.

#### Part Two: Postsurgery Surveys

The quantitative data of the case study was collected over a period of 12 weeks from June 2024 to September 2024. Theater nurses (including four participants from the interview) were surveyed following each RAS. They responded to task-specific items related to the surgery execution phase and to overarching scales on career sustainability and developmental needs. Variables and measures are displayed in Table 1. Approximately 28 RAS took place during this period, with a total of 44 returned questionnaires after surgery (79% response rate). Four data sets were excluded due to missing data. Four data sets were excluded because task variety was not quantifiable due to role switch or fluctuation during a procedure, resulting in n=36 data sets. Because some nurses had two or more da Vinci surgeries over the course of the study, seven participants completed the survey three times or more, and five participants completed it twice. In the current exploratory correlational analysis, the partially dependent data was treated as independent data points.

**Table 1.** Variables and measures of quantitative survey^f^.

Variable	Measures
Task variety	Number of specific tasks assigned to theater nurses during the surgery execution phase and was coded by authors following Rau et al [44].
Subjective workload	Three items from NASA-TLX^b^ [45] representing mental load, temporal load, and effort on a 15-point rating scale.
Subjective monotony	Two context-specific items on a 6-point rating scale, relating to underchallenge and boredom in DIN EN ISO^c^ 10075‐1 [46].
Perceived application of nontechnical cognitive skills (SA^d^ I-III)	Perceived application of nontechnical cognitive skills, measured by behaviorally anchored items adopted from established behavioral observation scales by Schreyer et al [30]. SA I: perceiving, SA II: understanding, SA III: projecting.
Perceived application of technical skills	Single-item, “I was able to apply my skills and abilities to the surgical process” on a 6-point rating scale.
Perceived career sustainability	Participants were asked to reflect: “Imagine that the surgery evaluated here would be a typical day at work for future theatre nurses. In light of this, how do you rate the following career related statements?.” Following De Vos et al [7] 3 indicators of sustainable careers were reflected (satisfaction, productivity, and health) using single items.
Growth need	Six items from the JDS^e^ originally developed by Hackman and Oldham [47].

a Note. All items are accessible in Multimedia Appendix 1

b NASA-TLX: NASA Task Load Index.

c DIN EN ISO: Deutsches Institut für Normung Europäische Norm International Organization for Standardization.

d SA: situational awareness.

e JDS: Job Diagnostic Survey.

### Ethical Considerations

The study adheres to ethical and legal requirements. Participation was voluntary, and informed consent was collected anonymously prior to participation. Participants received no form of compensation for their partaking in both parts of the study. Both parts of the study received prior ethical approval from the hospital’s Clinical Ethics Committee (CEC) and the Staff Representative Council. The study complies with the General Data Protection Regulation (GDPR); participants’ privacy and confidentiality of their data were maintained.

## Results

### Part One: Qualitative Data From the Focus Group Interview

#### Question 1

Nurses described varied experiences with workload, monotony, and skill application throughout RAS. During the phase of surgery execution (primary task), some reported a notably lower workload, largely due to the assistant surgeon taking over instrument handling. This reduction in engagement led to feelings of monotony and even boredom among theater nurses, who described this phase as repetitive. The reduction of involvement through instrument handling was associated with decreased SA. This can be seen as problematic since in RAS, nontechnical skills such as quick decision-making, cooperation, and quick adaptation to technical difficulties were noted as crucial. One aspect from the interview should be emphasized here and is reflected in the first implication at the end of this paper. It concerns the different evaluation of preparatory and concluding tasks (secondary tasks) in relation to the actual core task of executing the surgery. The monotony reported during surgery execution contrasted with heightened demands in surgery preparation and surgery conclusion. In these phases, staff often faced time pressures, especially in coordinating with anesthesia teams, whose shorter patient preparation times for RAS increase the demand on theater nurses. In response, nurses expressed a desire for additional support personnel. The lack of such support frequently led to overload, especially after undocking the robotic system, when multiple tasks needed immediate attention.

#### Question 2

Nurses also expressed complex views on how their involvement with RAS influences their job and career projections. The initial enthusiasm for working with advanced technology was reported to fade over time, with some indicating frustration due to the monotony during surgery execution. Additionally, although there was no concern over potential job replacement by the robotic system, questions were raised about future staffing needs: for example, whether two surgical nurses would remain necessary per RAS procedure. The lack of substantial, fulfilling tasks in RAS has led to a sense of diminished responsibility among some staff, with a desire for roles that offer more significant nontechnical skill applications and career development opportunities. While physical demands were not seen as problematic, some participants reported experiencing psychological stress due to workload peaks in phases of surgery preparation and surgery conclusion. The potential for increased productivity was noted, especially if procedural bottlenecks, such as moving and preparing the robotic system, were addressed. Some staff members mentioned actively trying to reduce their involvement in RAS by transitioning to hospitals with lower frequencies in order to gain enough variety in their schedules to prevent RAS routine monotony.

### Part Two: Quantitative Data From Postsurgery Surveys

Table 2 displays the nonparametric correlations (Spearman ρ) between the variables.

**Table 2.** Correlations (Spearman rho) among and descriptive statistics of case study variables^a^.

Variables	Task variety	Perceived workload	Perceived monotony	Nontech SA^b^ I	Nontech SA II	Nontech SA III	Nontech skill mean	Technical skill	Satisfaction	Productivity	Health	Growth need
Task level												
Task variety^c^												
ρ	1	0.13	–0.40^d^	0.43^d^	0.39^d^	–0.01	0.46^e^	0.39^d^	0.04	0.27	0.37^d^	–0.01
*P* value	֫—^f^	.44	.02	.008	.02	.98	.005	.02	.81	.12	.03	.95
Perceived workload^g^												
ρ	0.13	1	–0.50^e^	0.60^h^	0.21	–0.26	0.40^d^	0.08	0.13	0.17	0.47^e^	–0.48^e^
*P* value	.44	—	.002	<.001	.22	.13	.02	.65	.44	.32	.004	.003
Perceived monotony^i^												
ρ	–0.40^d^	–0.50^e^	1	–0.75^h^	–0.26	–0.07	–0.53^h^	–0.27	–0.08	–0.28	–0.4^d^	0.31
*P* value	.02	.002	—	<.001	.13	.70	<.001	.11	.64	.10	.01	.06
Nontech SA I^j^												
ρ	0.43^d^	0.60^h^	–0.75^h^	1	0.38^d^	0.09	0.75^h^	0.44^e^	0.10	0.26	0.50^e^	–0.34^d^
*P* value	.008	<.001	<.001	—	.02	.60	<.001	.007	.58	.13	.002	.04
Nontech SA II^k^												
ρ	0.39^d^	0.21	–0.26	0.38^d^	1	0.39^d^	0.85^h^	0.60^h^	0.13	0.15	0.42^d^	–0.48^e^
*P* value	.02	.22	.13	.02	—	.02	<.001	<.001	.45	.39	.01	.003
Nontech SA III^l^												
ρ	–0.01	–0.26	–0.07	0.09	0.39^d^	1	0.41^d^	0.59^h^	0.23	0.31	0.05	–0.09
*P* value	.98	.13	.70	.60	.02	—	.01	<.001	.17	.07	.77	.61
Nontech skill mean^m^												
ρ	0.46^e^	0.40^d^	–0.53^d^	0.75^h^	0.85^h^	0.41^d^	1	0.69^d^	0.15	0.28	0.57^h^	–0.48^e^
*P* value	.005	.02	<.001	<.001	<.001	.01	—	<.001	.39	.10	<.001	.003
Technical skill^n^												
ρ	0.39^d^	0.08	–0.27	0.44^e^	0.60^h^	0.59^h^	0.69^h^	1	0.28	0.33^d^	0.30	–0.30
*P* value	.02	.65	.11	.007	<.001	<.001	<.001	—	.10	.048	.08	.07
Career level												
Satisfaction^o^												
ρ	0.04	0.13	–0.08	0.10	0.13	0.23	0.15	0.28	1	.83^h^	0.50^e^	–0.30
*P* value	.81	.44	.64	.58	.45	.17	.39	.10	—	<.001	.002	.08
Productivity^p^												
ρ	0.27	0.17	–0.28	0.26	0.15	0.31	0.28	0.33^d^	.83^h^	1	0.59^h^	–0.18
*P* value	.12	.32	.10	.13	.39	.07	.10	.048	<.001	—	<.001	.29
Health^q^												
ρ	0.37^d^	0.47^e^	–0.4^d^	0.50^e^	0.42^d^	0.05	0.57^h^	0.30	0.50^e^	0.59^h^	1	–0.42^d^
*P* value	.03	.004	.01	.002	.01	.77	<.001	.08	.002	<.001	—	.01
Growth need^r^												
ρ	–0.01	–0.48^e^	0.31	–0.34^d^	–0.48^e^	–0.09	–0.48^e^	–0.30	–0.30	–0.18	–0.42^d^	1
*P* value	.95	.003	.06	.04	.003	.61	.003	.07	.08	.29	.01	—

a n=36, variables: “Nontech” refers to perceived application of nontechnical cognitive skills during a surgery, “Technical skill” refers to perceived application of technical skills during a surgery, “Satisfaction”, “Productivity”, and “Health” correspond to perceived career sustainability.

b SA: Situational Awareness.

c Mean (SD): 3.2 (0.66).

d *P*<.05.

e *P*<.01.

f Not applicable.

g Mean (SD): 5.24 (2.77).

h *P*<.001.

i Mean (SD): 3.59 (1.82).

j Mean (SD): 4.43 (0.93).

k Mean (SD): 4.92 (0.97).

l Mean (SD): 4.81 (1.01).

m Mean (SD): 4.69 (0.69).

n Mean (SD): 3.31 (1.91).

o Mean (SD): 3.77 (1.07).

p Mean (SD): 3.72 (1.06).

q Mean (SD): 3.00 (1.22).

r Mean (SD): 4.70 (1.07).

#### Question 1

Correlates indicate a relationship between objective task variety of theater nurses and perceived application of professional skills. The more tasks involved, the lower perceived monotony (ρ=−0.40; *P*=.02), and the higher perceived application of nontechnical (ρ=0.46; *P*=.005) as well as technical skills (ρ=0.39; *P*=.02). A similar pattern emerges for subjectively perceived workload during the surgical task.

#### Question 2

Results indicate that higher objective task variety and perceived workload during a surgical task are related to more positive evaluation of health-related career prospects (for task variety: ρ=0.37; *P*=.03 and for workload: ρ=0.47; *P*=.004). Similarly, the higher the perceived application of nontechnical and professional skills during a surgery the more positive reported health and productivity related career prospects appear. Moreover, results show a relationship between individual growth need and the individual evaluation of task characteristics and perceived skill application. Participants with high growth need report lower workload during surgery (ρ=−0.48; *P*=.003) and less perceived application of nontechnical (ρ=−0.48; *P*=.003) and technical skills (ρ=−0.30; *P*=.07).

## Discussion

This study investigated how task-specific demands shape theater nurses’ perceived professional skill application during RAS (Question 1), and how these task-level changes relate to broader evaluations of career sustainability and vice versa (Question 2).

Qualitative results showed that while the surgical execution phase reduced workload, it also heightened monotony and lowered situation awareness, whilst pre- and postoperative preparations led to overload due to time pressure and insufficient staffing. The quantitative data confirmed that in the execution phase higher task variety correlated with higher application of nontechnical and technical skills (Question 1). Moreover, task design and skill application during RAS were linked to nurses’ career reflections in the qualitative data. The survey showed higher task variety and skill application during surgeries were correlated with long-term career sustainability evaluations. Moreover, career-related growth needs correlate with interindividual differences in STARA task evaluations (Question 2).

### Limitations of the Case Study

Firstly, it is important to note that the presented correlational data can only be interpreted to a limited extent because of data independence, context-specific unvalidated scales, and small sample size. Repeated observations from the same nurses were included deliberately, as task variety differs situationally across procedures, capturing meaningful within-person variation–, though ensuring fully distinct evaluations remains challenging. Spearman ρ was chosen as it evaluates monotonic relationships regardless of distributional properties [48] with more robust type I error control than Pearson *r* [49,50]. Furthermore, besides task variety and career-related needs, additional contextual factors (eg, duration of the surgery) may affect perceptions of monotony and skill application. However, for exploratory reasons, the data allows for cautious reflections regarding task-specific work design in the field of Human Factors (Question 1). A decrease in task variety during RAS correlates with monotony and reduced application of professional skills. As task variety fluctuates between units, it is the responsibility of surgical team leaders to create conditions which increase task variety through more possibilities to apply nontechnical and technical skills. Task designs with higher applications of nontechnical cognitive skills enable faster and more resilient responses in case of disruptions. Moreover, this contributes to the maintenance and development of individual skills as a requirement of humane work design [8,51].

Examining the relationships between task-level and career evaluations in RAS provides interdisciplinary insights (Question 2). It is evident that both the specific task design of RAS (eg, task variety) and the associated application of professional skills are related to individual evaluations of career prospects. Furthermore, interindividual differences must be considered, as variance in personal growth need covaries with different evaluations of task characteristics and career prospects [1].

The case study gives practical insights with high ecological validity but is empirically limited due to a small sample size, situationally changing context conditions, or changing team compositions. For example, the small number of nurses working with da Vinci in a hospital are surveyed and observed on multiple measurement points. However, depending on the work plan or the motivation to participate, the within measurements vary greatly and range from only one instance of participation to up to 4 sessions per person. This results in a small between sample with varying within measurement points. Moreover, this data is confounded through the complexity and dynamic of the work setting, such as personnel changes during a surgery or “outlier” situations which need to be coded and removed from the statistical evaluation. For this reason, STARA’s interdisciplinary Career Research requires quantitative multilevel methods that are robust against the violation of prerequisites, as well as a broad mix of qualitative and multivariate quantitative methods.

### Theoretical, Practical, and Methodological Implications

The following section outlines the implications for Human Factors and interdisciplinary perspectives. These implications extend beyond the specific application of RAS and are discussed more broadly under the framework of STARA.

#### Human Factors’ Perspectives to Support Skill Application in STARA Environments

From a Human Factors’ perspective, the study’s findings emphasize that skill application in RAS is not determined by technology implementation alone, but rather by how surgical workflows are specifically designed and managed within different units of a hospital. Distinct task configurations across RAS procedures shape task-specific demands and are directly related to theater nurses’ perceived application of their professional skills. This highlights that leadership-driven work design and task allocation decisions, rather than STARA itself, play a critical role in whether skills are maintained, reduced, or expanded. Even within the same technological environment, variations between organizational units can create differences in opportunities for professional skill application. The consequences affect both broader system resilience and individual career projections.

First, professional skill application is closely tied to resilience, particularly through the maintenance of SA. In this case study, preserving nontechnical skills is not limited to perceiving and understanding or monitoring procedural steps (SA levels I and II), but also includes the critical ability to anticipate future disruptions or complications (SA level III). This anticipatory skill is essential for adaptive responses during unexpected events and directly contributes to resilience and safety of work systems. Examples are grounded in both health care and sociotechnical team research. In health care, projecting future states is critical for the early identification of complications and safety threats, thereby strengthening patient safety [52]. Furthermore, within sociotechnical team research, SA extends beyond immediate procedural coordination to encompass risk awareness regarding technological vulnerabilities, system limitations, and potential disruptions introduced by technologies themselves. This broader perspective is particularly relevant in STARA environments, where human actors must continuously perceive, understand, and project technology-related risks across a wide range of tasks [53].

Secondly, ongoing automation of tasks initiates a decrease in middle skilled jobs, such as theater nurses, leading to the individual use of career transition strategies [4]. This very effect became evident in the qualitative part of the case study when nurses mentioned transitioning from high-volume RAS sites to hospitals with lower frequencies. To avoid these negative STARA effects, human-centered design principles should be explicitly considered during the implementation of technologies. For example, Parker and Grote [8] underline the importance of task-variety, as well as human autonomy, skill-use, social interaction, or feedback. These factors can contribute to shaping the workplace and, thus indirectly, the careers of workers in STARA contexts in a way that is sustainable, healthy, productive, and beneficial to personal development. For example, lower task-variety in one subtask may be compensated with additional primary or secondary tasks so that employees remain involved and can develop personal competencies. In the present case study, lower task-variety during surgery could be complemented by implementing mixed reality technologies in RAS to inform and involve nurses during inactive periods [54,55]. A prospective strategy would focus not only on skill training, but on fostering proactive work as well as task design. Providing competencies and resources for joint optimization of changing workplaces can foster the adaptation to STARA-induced changes [56]. Employees who perceive STARA as an opportunity, rather than a threat, are more likely to engage in job crafting [57], which may also support sustainable careers [5]. Designing human-centered STARA environments therefore requires preserving meaningful opportunities for both technical and nontechnical skill application to promote long-term career sustainability.

Consequently, task design should be understood as a strategic lever for fostering both sustainable careers and system resilience.

#### Interindividual Differences in Skill Application and Career-Related Needs Are Relevant for Evaluating STARA Work Systems

Human Factors as well as Career Research can benefit from a differential approach focusing on interindividual differences.

Human Factors Research should also consider that there will be interindividual variations in the evaluation of task-characteristics within a given STARA work environment. Evaluations of STARA work systems go beyond task characteristics (see Question 2b). In our case study, we found evidence that interindividual differences in individual growth needs correlate with subjective task evaluations (but not with the objective task variety). Nurses with high growth needs seemed to perceive generally lower workload and skill application than nurses with lower needs. Theoretically, the importance of individual motives on the assessment of work systems is not completely new (Hackman and Oldham [58] for work satisfaction, motivation, and turnover). STARA modifies task characteristics, but individual motives and interindividual differences in task evaluation are often neglected in Human Factors. Numerous motives and personality traits related to STARA work environments can be identified in organizational and Career Research, which may affect the evaluation of the system design in a specific work context (eg, need for autonomy or personal growth [38]). In this context, an interdisciplinary career perspective offers numerous starting points for Human Factors Research and theory development. Differential work design can be used to consider varying development needs based on these findings of interindividual differences in motives and traits.

For Career Research it is important to acknowledge that the perception of skill application in a particular task is highly individual. Beyond that, the interindividual perceptions also relate to the evaluation of indicators for sustainable careers [7]. Question 2a of our case study focused on perceived application of technical and nontechnical skills relevant to system resilience [3] and its link to career reflections. Nurses reporting higher application of role-related nontechnical skills reflected positively on career health, whereas perceived application of technical skills correlated with career productivity. This link between interindividual different perceptions of task-related skills and career evaluations should be considered in future Career Research as theories mention the complexity of objective environmental conditions and subjective evaluations resulting in a wide variety of individual reflections on whether one’s career is sustainable or not [7]. For future investigations, Human Factors offers systematic measures of technical and nontechnical skills [30] which can also be applied in practice for resilient task design, personnel development, and career counseling.

#### Intraindividual Changes Are a Significant Temporal Variable for Theory and Practice

Theories of career sustainability underline the importance of temporal dynamics and intraindividual variation over time from a methodological and conceptual standpoint [5,7,34].

In the short term, research on career sustainability may investigate the effects of time and quality of individual experiences with STARA technology. In this respect, Human Factors Research provides measures and evidence about user experience [59]. User experience does not only have a quantitative temporal focus (ie, the duration of experience with a STARA technology), but also a qualitative one. For example, quality depends on whether STARA experiences fulfill effort and performance expectations [12]. Thus, time and quality of user experience may add technology-related variables to the individual level of models on career sustainability, stimulating future research on STARA implementation.

In the long term, attitudes towards STARA may vary throughout an individual’s lifetime. As shown in gerontological research [60], goals related to personal growth and new experiences decrease with age. This variable is related to growth needs and was negatively correlated with perceived career sustainability in our study. Future research may investigate how shifts in personal goals or other motives impact career evaluations, especially in STARA environments.

#### Human Factors Task Characteristics Expand Models of Career Sustainability

STARA includes an overwhelming variety of technical applications with different implications for workers’ career sustainability across diverse contexts. For example, Chin et al [5] focus on manufacturing workers facing full automation of the entire work task correlated with job insecurity and fear of job losses. In contrast, in our case study of RAS, the automation affected one specific subtask regarding SA. The two examples differ essentially in the extent of STARA-induced changes and subsequent career assessments but become comparable on task-level characteristics. Present conceptual frameworks of career sustainability [5] could benefit from expanding the individual and organizational levels to a task-level with different variables such as LoA, task type, or task variety.

First, LoA [61] indicates the degree of autonomy with which STARA technology performs tasks independently from human control. Low degrees of technical autonomy, such as in our RAS example, can have very different effects on career sustainability compared to high degrees of automation, where machines generate and implement decisions without human involvement. Second, automation through STARA may affect different task types like monitoring, planning, decision-making, or execution [11]. In one context, automation can be related to an executing task in manufacturing [5]. In another context, STARA might automate a monitoring task, leaving decision and execution tasks to humans with different consequences for career evaluations. Third, task-variety can be used to investigate STARA-induced changes between or within contexts. As shown in the case study, nurses’ evaluations of career sustainability can even differ when working in the same context with the same technology but in different surgical work units. Nurses with higher objective task variety seemed to reflect generally more positively about anticipated health, one indicator of sustainable careers. Furthermore, task-level analysis also involves the composition of primary and secondary tasks. In the case study, we focused on the variety of tasks nurses performed during the primary task of surgery execution. In addition to this core task, there are preliminary and concluding secondary tasks that might shift in their task characteristics due to STARA and thus influence career evaluations.

The task-level variables mentioned could be further supplemented, for example, by STARA-relevant team-level variables such as team cohesion or team trust [12]. As has been requested in previous studies on sustainable careers [5], task and team-level variables from Human Factors could enrich the framework as relevant predictors, mediators, or moderators, and inspire future studies.

#### Interdisciplinary Perspectives on STARA Require Conceptual and Methodological Clarity

The former 4 implications display valuable synergies of interdisciplinary research by integrating person, task, and technology characteristics into frameworks of sustainable career as well as Human Factors research. This added value was also highlighted in the case study, which will now be supplemented by conceptual and methodological desiderata for future research.

When interdisciplinary concepts are integrated into models, it must be ensured that variables with the same name do not represent different content or operationalization. In our case study, the perceived application of technical and nontechnical skills was a variable connecting Human Factors with Career Research. Both disciplines have an extensive body of research on skill-related concepts, measures, and models (eg, recruitment diagnostic, and lifelong learning in Career Research; skills for system resilience, and problem solving in Human Factors). However, we were faced with the challenge that even within the discipline of Human Factors, researchers applied terminologies and definitions heterogeneously, which calls urgently for conceptual clarifications. For example, some authors refer to nontechnical skills as skills [27] some as competencies [62]. This issue, referred to as Jingle-Jangle-Fallacy, hinders STARA-related research, because interdisciplinarity increases language diversity [63].

Besides conceptual clarity, our study underlines the careful distinction between objective and subjective evaluations on a measurement level. For example, it is necessary to carefully distinguish between the objective expression of professional skills in a STARA context and the subjective self-evaluations as conceptualized in this study. Consequently, for fruitful interdisciplinary research, it is crucial that concepts, definitions, and operationalization are clearly described and that future work identifies existing inconsistencies (for methods see [63]).

### Conclusion

This paper highlights the importance of conceptually and empirically linking task characteristics to career sustainability in health care-related STARA work contexts and beyond. It is the way new technologies are implemented into existing work processes, and not technology itself, that changes task characteristics. This, in turn, affects both short- and long-term employee career experiences in the health-care sector. Research on sustainable careers in particular can benefit from the expansion of existing theories to include highly task- and context-specific variables that can explain inter- and intraindividual differences in career evaluation. Thus, cross-disciplinary insights can and should enrich Career Research’s theoretical models, empirical investigations, and practical interventions with constructs and measures.

## Supplementary material

10.2196/87735 Multimedia Appendix 1 Measures from Part Two of the Case Study: Quantitative Data from Postsurgery Surveys.

## Abbreviations

CEC: Clinical Ethics Committee
GDPR: General Data Protection Regulation
LoA: Level of Autonomy
RAS: robot-assisted surgery
SA: Situational Awareness
STARA: Smart Technology, AI, robotics, and algorithms
