# Supplementary material for: Shifting Skills in Robot-Assisted Surgery: Case Study With Interdisciplinary Perspectives for Human Factors and Career Research
Source: JMIR Hum Factors. 2026 Sep 22;13:e87735. doi: 10.2196/87735 (PMC13600337; doi:10.2196/87735)
Supplement: Supplementary file 1 [file humanfactors-v13-e87735-s001.docx]

**Measures from Part Two of the Case Study:**

**Quantitative Data from Post-Surgery Surveys**

(1) Task variety.

The number of specific tasks assigned to theatre nurses during the surgery execution phase was used as an objective indicator. In expert interviews, key tasks in different departments (urological and visceral) were identified and counted [1]. In some departments, task variety included two tasks (handing over instruments, adjusting da Vinci instruments); in others, three tasks (additional participation, such as splinting the urethra by moving the catheter to assist during anastomosis) up to four tasks for circulators (e.g., documenting surgery materials, communication during surgery, preparing for the upcoming surgeries, photographic documentation).

(2) Subjective Workload.

This variable was operationalised by the mean of three items from NASA-TLX [2] representing mental load, temporal load and effort on a 15-point rating scale with endpoints *very low* to *very high*. German translations were taken from Seifert [3]:

Mental Load: “How much mental and perceptual activity was required (e.g., thinking, deciding, calculating, remembering, looking, searching, etc.)? Was the task easy or demanding, simple or complex, exacting or forgiving?”

Temporal Load: “How much time pressure did you feel due to the rate or pace at which the tasks or task elements occurred? Was the pace slow and leisurely or rapid and frantic?”

Effort: “How hard did you have to work (mentally and physically) to accomplish your level of performance?“

(3) Perceived monotony.

Monotony was measured using two context-specific items on a 6-point rating scale with endpoints (1 = *not at all* to 6 = *very much*). Items relate to underchallenge and boredom taken from the definition of monotony in DIN EN ISO 10075-1 [4].

“I felt underchallenged during the console phase.”

“I felt bored during the console phase.”

(4) Perceived application of non-technical cognitive skills.

This was measured by behaviourally anchored items adopted from established behavioural observation scales by Schreyer et al. [5]. The focus was on non-technical cognitive skills representing three different levels of situational awareness during the surgery task. Each item described two situations, A and B. Participants rated, what situation was experienced during the surgery on a 6-point rating scale: 1 = *Exactly like A*, 2 = *similar to A*, 3 = *tends towards A*, 4 = *tends towards B*, 5 = *similar to B*, 6 = *exactly like B*. Responses were recoded that higher values indicate high levels of situational awareness.

SA I “Noticing and Perception” was measured with three items

Item Example. “Which of the two situations, A or B, most closely matched your experience? / A = I was able to perceive changes, anomalies, and deviations in the surgical process. / B = I was distracted and couldn’t always perceive changes, anomalies, and deviations.”

SA II “Understanding” was measured with three items.

Item example: “Which of the two situations, A or B, most closely matched your experience? / A = I was always clear about whom to address with requests or questions. / B = I was sometimes unsure about whom to address with requests or questions.”

SA III “Projecting” was measured with a single-item.

Item: “Which of the two situations, A or B, most closely matched your experience? / A = I was able to anticipate well/in time if additional materials would be needed or if agreements needed to be made. / B = I had difficulty predicting if additional materials would be needed or if agreements would need to be made.”

(5) Perceived application of technical skills.

This was measured using a single-item (“I was able to apply my skills and abilities to the surgical process”) on a 6-point rating scale with endpoints 1 = *not at all* and 6 = *very much*.

(6) Perceived Career Sustainability.

Following De Vos et al. [6] three indicators of sustainable careers were reflected using single-items on a 5-point direct response rating scale (1 = *Yes* to 5 = *No*). Responses were recoded that high scores indicate more positive evaluations.

Before the ratings, participants were asked to “imagine that the surgery evaluated here would be a typical day at work for future operating room nurses. In light of this, how do you rate the following career facets?”

Item Satisfaction: “The career is satisfying and interesting.”

Item Productivity: “Skills and abilities are developed to improve performance.”

Item Health: “The career promotes physical and mental well-being.”

(7) Growth Need.

Employees’ growth need was operationalised using six items from the Job Diagnostic Survey (JDS) originally developed by Hackman and Oldham [7]. German items were taken from van Dick et al. [8] and rated on a 5-point scale with endpoints (1 = *Yes* to 5 = *No*). Responses were recoded so that high scores indicate high growth need.

How much you personally would like to have ...

Item 1: “Stimulating and challenging work.”

Item 2: “Chances to exercise independent thought and action in my job.”

Item 3: “Opportunities to learn something new at work.”

Item 4: “Opportunities to be creative and imaginative in my work.”

Item 5: “Opportunities for personal growth and development in my job.”

Item 6: “A sense of worthwhile accomplishment in my work.”

References

1. Rau R, Schweden F, Hoppe J, Hacker W. Verfahren zur Tätigkeitsanalyse und -gestaltung bei mentalen Arbeitsanforderungen (TAG-MA). Kröning, Germany: Asanger; 2021. ISBN:9783893346486
2. Hart SG, Staveland, LE. Development of NASA-TLX (Task Load Index): results of empirical and theoretical research. In: Hancock PA, Meshkati N, editors. Advances in Psychology: Human Mental Workload. Vol 52. North-Holland: Elsevier; 1988:139-183. ISBN:9780444703880
3. Seifert K. Evaluation multimodaler Computer-Systeme in frühen Entwicklungsphasen: Ein empirischer Ansatz zur Ableitung von Gestaltungshinweisen für multimodale Computer-Systeme. Dissertation. Technische Universität Berlin, Germany; 2002. doi: 10.14279/depositonce-530
4. DIN EN ISO 10075-1. Ergonomic principles related to mental workload - part 1: general issues and concepts, terms and definitions (ISO 10075-1:2017). German version EN ISO 10075-1:2017*.* Berlin, Germany: DIN Media; 2018. doi:10.31030/2654667
5. Schreyer J, Koch A, Herlemann A, et al. RAS-NOTECHS: validity and reliability of a tool for measuring non-technical skills in robotic-assisted surgery settings. Surg Endosc. 2022;36(3):1916-1926. doi:10.1007/s00464-021-08474-2
6. De Vos A, Van Der Heijden BIJM, Akkermans J. Sustainable careers: towards a conceptual model. J Vocat Behav. 2020;117:103196. doi:10.1016/j.jvb.2018.06.011
7. Hackman JR, Oldham GR. (1980). Work redesign. Boston, MA: Addison-Wesley; 1980. ISBN: 9780201027792
8. van Dick R, Schnitger C, Schwartzmann-Buchelt C, Wagner U. Der Job Diagnostic Survey im Bildungsbereich, Zeitschrift für Arbeits- und Organisationspsychologie*.* 2001;45(2):74-92. doi:10.1026/0932-4089.45.2.74
